# Prediction of In-Hospital Respiratory Support Among Children Aged 2–59 Months Hospitalized with Pneumonia in Southern Vietnam: A Retrospective Cohort Study

**DOI:** 10.3390/jcm15072490

**Published:** 2026-03-24

**Authors:** Thi Van Vo, Phuong Minh Nguyen, Dien Tri Lu, Thanh Huy Ong, Tri Duc Nguyen, Dien Minh Thai, Duc Hoang Minh Tran

**Affiliations:** 1 Department of Pediatrics, Faculty of Medicine, Can Tho University of Medicine and Pharmacy, No. 179 Nguyen Van Cu Street, Tan An Ward, Can Tho 900000, Vietnam; vvthi@ctump.edu.vn (T.V.V.); nmphuong@ctump.edu.vn (P.M.N.); 2 Internal Medicine Program, Department of General Nursing, Faculty of Nursing and Medical Technology, Can Tho University of Medicine and Pharmacy, No. 179 Nguyen Van Cu Street, Tan An Ward, Can Tho 900000, Vietnam; lutridien@ctump.edu.vn; 3 Can Tho Children’s Hospital, No. 345 Nguyen Van Cu Street, An Binh Ward, Can Tho 900000, Vietnam; drhuythanh146@gmail.com (T.H.O.); ngductri27@gmail.com (T.D.N.); 4 Faculty of Medicine, Nam Can Tho University, No. 168 Nguyen Van Cu Street, An Binh Ward, Can Tho 900000, Vietnam; dien211208@student.nctu.edu.vn

**Keywords:** child, pneumonia, respiratory support, risk prediction, neutrophil-to-lymphocyte ratio, logistic regression, Vietnam

## Abstract

Respiratory support requirement among children hospitalized with pneumonia is a key marker of disease severity and resource needs, yet scalable risk stratification tools for routine hospital settings in Southern Vietnam remain limited. **Background**: This study aimed to develop and evaluate clinical and laboratory-based multivariable models to predict respiratory support requirement in children under five hospitalized with pneumonia, using a routine care dataset. **Methods**: We conducted a retrospective cohort study conducted at a tertiary pediatric hospital in Southern Vietnam (July 2024–November 2025), children aged 2–59 months hospitalized with pneumonia were included after predefined exclusions. The outcome was the maximum (worst) level of respiratory support required during hospitalization (oxygen therapy, CPAP, or invasive mechanical ventilation), analyzed as a binary endpoint (any support vs. none) for model development. Candidate predictors included bedside clinical variables (age < 12 months, malnutrition, recurrent pneumonia, cyanosis, tachypnea, chest indrawing) and complete blood count-derived inflammatory indices. Univariable logistic regression was used for crude associations. Two multivariable logistic regression models were built: Model 1 (clinical-only) and Model 2 (clinical + neutrophil-to-lymphocyte ratio [NLR]; primary). Discrimination was assessed using area under the ROC curve (AUC), and calibration was evaluated using the Hosmer–Lemeshow test and observed-to-expected (O:E) ratio. **Results**: A total of 1797 children were included; 154 (8.6%) required respiratory support. In the primary model, independent predictors were age < 12 months (aOR 2.57, 95% CI 1.69–3.92), malnutrition (aOR 4.33, 2.56–7.33), recurrent pneumonia (aOR 1.82, 1.18–2.81), cyanosis (aOR 24.02, 7.41–77.87), chest indrawing (aOR 4.19, 2.73–6.43), and higher NLR (per 1 unit: aOR 1.49, 1.38–1.60), while tachypnea was not independently associated after adjustment. Discrimination improved from Model 1 (AUC 0.754) to Model 2 (AUC 0.840; 95% CI 0.806–0.874). At the optimal probability cut-off (0.122), Model 2 achieved sensitivity 66.2%, specificity 86.2%, PPV 31.1%, NPV 96.5%, and accuracy 84.5%. Calibration was acceptable (Hosmer–Lemeshow *p* = 0.662; O:E = 1.00). **Conclusions**: A simple clinical model strengthened by NLR provided good discrimination and calibration for predicting respiratory support requirement among children under-five hospitalized with pneumonia in Southern Vietnam. This approach may support early triage, prioritization of monitoring intensity, and escalation readiness in resource-constrained settings, although external validation is warranted.

## 1. Introduction

Pneumonia remains one of the leading causes of morbidity and hospitalization among children under five years of age worldwide, particularly in low- and middle-income countries (LMICs) [1,2,3]. Despite substantial progress in child survival over recent decades, lower respiratory tract infections, particularly pneumonia, continue to represent a major cause of global morbidity and mortality among children younger than five years of age, with the highest burden observed in low- and middle-income countries [2,4]. In the Western Pacific region, pneumonia is recognized as a priority childhood illness in clinical management guidelines, and hospital-based studies from Vietnam have demonstrated substantial variation in disease severity and clinical outcomes among children admitted with pneumonia [5,6,7].

Among hospitalized children with pneumonia, the presence of severe clinical features and the subsequent need for respiratory support are associated with worse clinical outcomes, including increased risk of intensive care admission and mortality [8,9]. Early identification of children with pneumonia who are at risk of severe disease is important to support timely clinical decision-making and appropriate management, particularly in resource-limited settings [3,9].

Current international guidelines, including those from the World Health Organization and the British Thoracic Society, emphasize clinical signs such as tachypnea, chest indrawing, cyanosis, and hypoxemia for identifying severe pneumonia and guiding decisions regarding hospital admission and intensified clinical management [3,10,11]. Evidence from systematic reviews indicates that individual clinical signs used in isolation have highly variable sensitivity and specificity for the diagnosis of pneumonia in young children, underscoring the limitations of relying on single clinical indicators [12]. This has prompted growing interest in multivariable risk stratification approaches that integrate multiple clinical and laboratory features to improve predictive accuracy.

In recent years, prediction models for pediatric pneumonia severity and high-acuity outcomes have increasingly been explored using both traditional regression-based methods and machine learning techniques [13,14]. These models aim to support early decision-making in emergency and inpatient settings by estimating the probability of deterioration rather than relying solely on dichotomous severity classifications. Hospital-based studies from low- and middle-income settings, including Vietnam, have described substantial heterogeneity in disease severity, comorbidity profiles, and clinical outcomes among children hospitalized with pneumonia, highlighting important contextual differences compared with high-income settings [5].

In addition to bedside clinical assessment, laboratory markers such as C-reactive protein have been evaluated for their diagnostic value in children with suspected pneumonia; however, evidence suggests that CRP alone has limited accuracy and should not be used as a standalone indicator [15]. In contrast, complete blood count (CBC)–derived indices are inexpensive, widely available, and rapidly obtained. The neutrophil-to-lymphocyte ratio (NLR) has emerged as a pragmatic inflammatory marker associated with disease severity and adverse outcomes in pediatric respiratory infections, including community-acquired pneumonia [16]. By reflecting the balance between innate immune activation and lymphocyte suppression, NLR offers biological plausibility as a marker of systemic inflammatory stress relevant to respiratory deterioration.

For prediction research to be clinically credible, transparent reporting and appropriate evaluation of discrimination and calibration are essential. The TRIPOD statement and its recent extension, TRIPOD + AI, provide guidance for reporting multivariable prediction models developed using regression or machine learning approaches [17,18]. In addition, the PROBAST framework highlights key sources of bias and applicability concerns in prediction model studies, underscoring the importance of careful model specification and validation, particularly for single-center retrospective analyses [19,20].

Against this background, we conducted a retrospective cohort study of children aged 2–59 months hospitalized with pneumonia at a tertiary pediatric hospital in southern Vietnam between July 2024 and November 2025. The objectives were to (1) describe baseline clinical and laboratory characteristics according to respiratory support status, and (2) develop and internally evaluate multivariable prediction models - combining bedside clinical features with CBC-derived NLR—to predict respiratory support requirement, with formal assessment of discrimination and calibration to inform future external validation and clinical implementation.

## 2. Materials and Methods

### 2.1. Study Design and Setting

We conducted a retrospective cohort study at a tertiary pediatric hospital in southern Vietnam from July 2024 to November 2025. The study used routinely collected clinical data extracted from medical charts and hospital electronic databases. We aimed to develop and internally evaluate multivariable models to predict in-hospital respiratory support requirement among children hospitalized with pneumonia.

### 2.2. Participants

#### Eligibility Criteria

We included children aged 2–59 months who were hospitalized with pneumonia during the study period. Pneumonia was defined using WHO-aligned clinical criteria, i.e., cough and/or difficulty breathing (with or without fever) accompanied by at least one of the following:

Age-specific tachypnea (≥50 breaths/min for 2–<12 months; ≥40 breaths/min for 12–59 months), and/or

Lower chest wall indrawing, and/or

Abnormal lung auscultation (e.g., crackles/crepitations or wheeze) documented at admission.

### 2.3. Exclusion Criteria

Children were excluded if they were aged outside 2–59 months, had an uncertain pneumonia diagnosis, had missing key variables required for analysis, or had major chronic cardiopulmonary conditions likely to confound respiratory status. For children with multiple admissions during the study period, only the first eligible admission was included, so the analytic cohort comprised unique children.

#### Study Sample

A total of 1797 unique children (first eligible admission) were included in the final analytic cohort.

### 2.4. Outcome Definition

The primary outcome was the maximum (worst) level of respiratory support required at any time during the index hospitalization, operationalized as a binary variable (0 = no respiratory support; 1 = any respiratory support). Respiratory support included supplemental oxygen therapy, non-invasive ventilation (CPAP), and invasive mechanical ventilation (IMV) as documented in the medical record. Importantly, all candidate predictors were assessed at admission (triage timepoint), whereas the outcome was ascertained over the entire hospitalization to capture subsequent clinical deterioration and escalation of respiratory care. In our respiratory ward setting, respiratory support typically follows an escalation continuum (oxygen → CPAP → IMV); therefore, defining the endpoint using the maximum level reached provides a severity-oriented and pragmatic outcome relevant for escalation readiness and resource planning in routine care. This endpoint was operationalized and analyzed as a binary outcome (any respiratory support vs. none) for the primary multivariable logistic regression model.

### 2.5. Candidate Predictors

Predictors were selected a priori based on clinical plausibility and routine availability at admission. The candidate predictors included:

#### 2.5.1. Clinical Predictors (At Admission)

Age category: <12 months vs. ≥12 months

Sex

Malnutrition (binary), based on documented nutritional assessment using WHO 2006 growth standards (weight-for-age and/or height-for-age as recorded in clinical practice)

Recurrent pneumonia (binary), defined as ≥2 episodes/year or ≥3 lifetime episodes per caregiver report/record

Signs of respiratory compromise documented at admission (binary):

Cyanosis

Tachypnea (as defined above)

Chest indrawing

Coding convention used in analyses: 0 = No/Absent, 1 = Yes/Present for all binary predictors.

#### 2.5.2. Laboratory Predictors (At Admission)

Complete blood count (CBC) indices were extracted when available. The main laboratory predictor for the primary model was:

Neutrophil-to-lymphocyte ratio (NLR) (continuous), calculated as neutrophil%/lymphocyte% from admission CBC.

To reduce collinearity, NLR was used instead of including neutrophil% and lymphocyte% simultaneously in the same multivariable model.

CRP and other biomarkers were described where available; however, predictors with substantial missingness (e.g., CRP) were not prioritized for the primary model to avoid loss of sample size and selection bias.

### 2.6. Data Sources and Data Collection

Data were extracted retrospectively from hospital medical records and electronic databases using a standardized data abstraction form. Extracted elements included demographics, admission clinical signs, comorbidities relevant to exclusion criteria, CBC results, respiratory support interventions during hospitalization, ICU transfer, length of stay, and discharge outcomes. Quality checks included range checks, consistency checks between related fields (e.g., respiratory support orders vs. nursing flow sheets), and review of outliers. Respiratory support interventions were ascertained using physician orders and nursing flow sheets (oxygen delivery records, CPAP settings, and ventilation documentation) to improve measurement consistency. Discrepancies between orders and nursing documentation were resolved by chart review. Data were abstracted using a standardized form, and all extracted datasets were de-identified prior to analysis.

### 2.7. Statistical Analysis

All analyses were performed using IBM SPSS Statistics (version 27; IBM Corp., Armonk, NY, USA).

AI-assisted language editing was used to improve grammar, clarity, and readability; all scientific content, analyses, and final wording were reviewed and approved by the authors.

Descriptive analysis: Continuous variables were summarized as median (IQR) and compared using the Mann–Whitney U test. Categorical variables were summarized as n (%) and compared using χ^2^ test or Fisher’s exact test when cell counts were small (e.g., cyanosis). 

Outcome coding. Candidate predictors were assessed at admission (triage timepoint). The outcome was defined as the maximum (highest-acuity) respiratory support required at any time during the index hospitalization and was coded as a binary variable (1 = any respiratory support; 0 = none) for logistic regression analyses.

Univariable association: Crude associations with the outcome were estimated using univariable logistic regression, reported as odds ratios (OR) with 95% confidence intervals (CI).

Multivariable model development: two prespecified logistic regression models were fitted:

Model 1 (Clinical-only): age group, malnutrition, recurrent pneumonia, cyanosis, tachypnea, chest indrawing (and sex if prespecified).

Model 2 (Clinical + NLR; primary): all Model 1 predictors plus NLR (continuous).

Model performance:

Discrimination: AUC (ROC) with 95% CI.

Classification: Sensitivity, specificity, PPV, NPV, and accuracy at the optimal cut-off determined by the Youden index from the ROC curve of predicted probabilities.

Calibration: Hosmer–Lemeshow goodness-of-fit test (deciles of predicted risk) and observed-to-expected (O:E) ratio

Significance threshold: Two-sided *p* < 0.05.

### 2.8. Ethical Considerations

The study was conducted in accordance with the Declaration of Helsinki and relevant institutional regulations. This retrospective cohort study used routinely collected clinical data extracted from medical records; no additional procedures or interventions were performed for research purposes. Ethical approval was granted by the Ethics Committee in Biomedical Research, Can Tho University of Medicine and Pharmacy (Approval No. 24.304.HV/PCT-HĐĐĐ; 28 June 2024; expedited review), including a waiver of informed consent due to the retrospective nature of the study and minimal risk to participants. All datasets were coded/de-identified prior to analysis, and confidentiality was maintained throughout data handling and reporting. The study was conducted at Can Tho Children’s Hospital, Vietnam.

## 3. Result

### 3.1. Study Cohort and Outcome Frequency

**Figure 1:**
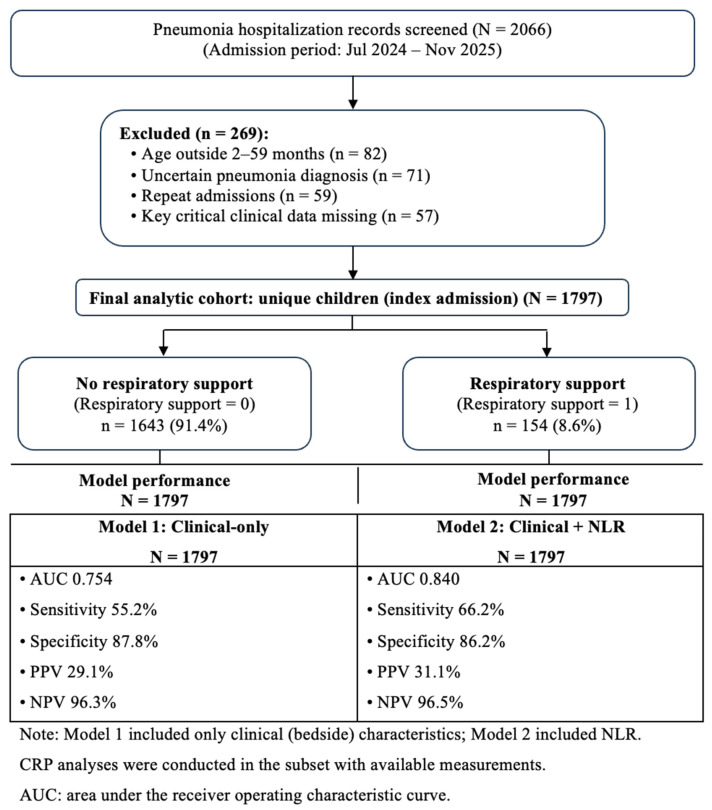
Study flowchart. Note: Respiratory support breakdown (*n* = 154). Initial modality: oxygen therapy 150/154 (97.4%) and CPAP 4/154 (2.6%); no child was intubated as the initial modality. Maximum (worst) level reached during hospitalization: oxygen only 114/154 (74.0%), CPAP without intubation 24/154 (15.6%), and invasive mechanical ventilation (IMV) 16/154 (10.4%). Among IMV cases, 12 escalated directly from oxygen to IMV without CPAP, while four escalated from CPAP to IMV.

During the study period, 2066 hospitalization records of pediatric pneumonia were screened. After excluding 269 records (age outside 2–59 months, uncertain pneumonia diagnosis, repeat admissions [only the first eligible admission per child was retained], and missing key clinical data), 1797 children were included in the final analysis. Of these, 154 (8.6%) required respiratory support during hospitalization, while 1643 (91.4%) did not (Figure 1). Among the 154 children who required respiratory support, the initial modality was oxygen therapy in 150/154 (97.4%) and CPAP in 4/154 (2.6%); no child was intubated as the initial modality. When classified by the maximum (worst) respiratory support level reached during hospitalization, 114/154 (74.0%) received oxygen only, 24/154 (15.6%) required CPAP without intubation, and 16/154 (10.4%) required invasive mechanical ventilation. Of the 16 IMV cases, 12 escalated directly from oxygen to IMV without CPAP, while four escalated from CPAP to IMV.

### 3.2. Baseline Characteristics by Respiratory Support Status 

Children requiring respiratory support were younger (median age 12.5 vs. 17.0 months, *p* = 0.012) and had a markedly higher prevalence of malnutrition (24.7% vs. 5.2%) and recurrent pneumonia (35.7% vs. 15.6%) (both *p* < 0.001). Key clinical signs of respiratory compromise were substantially more common in the support group, including cyanosis (11.7% vs. 0.4%; Fisher’s exact *p* < 0.001), tachypnea (47.4% vs. 29.8%; *p* < 0.001), and chest indrawing (53.2% vs. 23.2%; *p* < 0.001). In laboratory profiles, the support group had higher CRP, higher neutrophil percentage, lower lymphocyte percentage, and higher NLR (all *p* < 0.001), whereas WBC and MPV did not differ meaningfully (*p* = 0.975 and 0.088, respectively). CRP was available for 909/1797 children (missing 888), and comparisons involving CRP should be interpreted in light of this missingness (Table 1).

### 3.3. Univariable Associations

In univariable logistic regression, the strongest crude predictors of respiratory support were cyanosis (OR 36.11, 95% CI 14.10–92.47), malnutrition (OR 6.00, 3.92–9.20), and chest indrawing (OR 3.77, 2.69–5.28) (all *p* <0.001). Age < 12 months was associated with nearly doubled odds of support (OR 1.99, 1.43–2.77), and recurrent pneumonia was also associated (OR 3.00, 2.10–4.28). Among laboratory predictors, NLR showed a strong association (OR 1.33 per 1 unit, 1.25–1.41; *p* < 0.001), while CRP had a smaller but significant effect (OR 1.08 per 10 mg/L, 1.02–1.15; *p* = 0.013; CRP available in 909 children). Male sex was not associated (OR 1.00; *p* = 0.991) (Table 2).

### 3.4. Multivariable Models (Primary Model = Clinical + Nlr)

In the clinical-only model (Model 1), cyanosis, chest indrawing, malnutrition, and recurrent pneumonia remained independent predictors of respiratory support (all *p* ≤ 0.007). Tachypnea was not independently associated after adjustment (*p* = 0.294), suggesting overlap with more specific severe signs (cyanosis and chest indrawing). Age < 12 months showed a borderline association in Model 1 (*p* = 0.091). After adding NLR (Model 2, primary), NLR remained independently associated with respiratory support (aOR 1.49 per 1 unit, 95% CI 1.38–1.60; *p* < 0.001), and the effect of age < 12 months became stronger (aOR 2.57, 1.69–3.92; *p* < 0.001). Other independent predictors in Model 2 included malnutrition (aOR 4.33), recurrent pneumonia (aOR 1.82), cyanosis (aOR 24.02), and chest indrawing (aOR 4.19) (Table 3).

### 3.5. Discrimination (Roc) and Calibration

**Figure 2:**
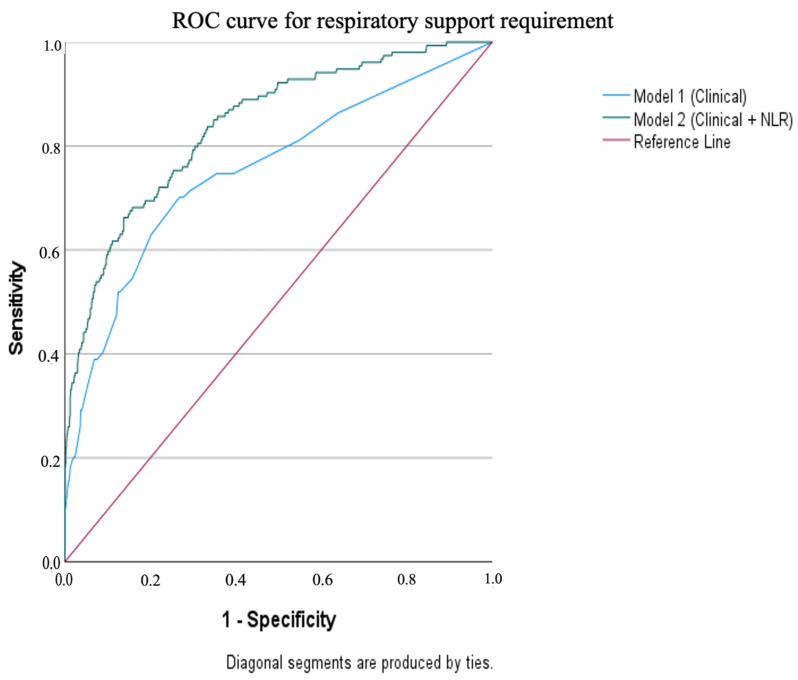
ROC curves of the multivariable models.

For single predictors, NLR provided the highest discrimination among laboratory indices (AUC 0.705), followed by neutrophil percentage (AUC 0.694) and CRP (AUC 0.670), while WBC and MPV performed poorly. Age and lymphocyte percentage had AUC values < 0.50, indicating inverse relationships (younger age and lower lymphocyte% associated with higher risk). The clinical-only model demonstrated good discrimination (AUC 0.754, 95% CI 0.706–0.797), which improved substantially after adding NLR (Model 2 AUC 0.840, 95% CI 0.805–0.873) (Table 4; Figure 2).

**Figure 3:**
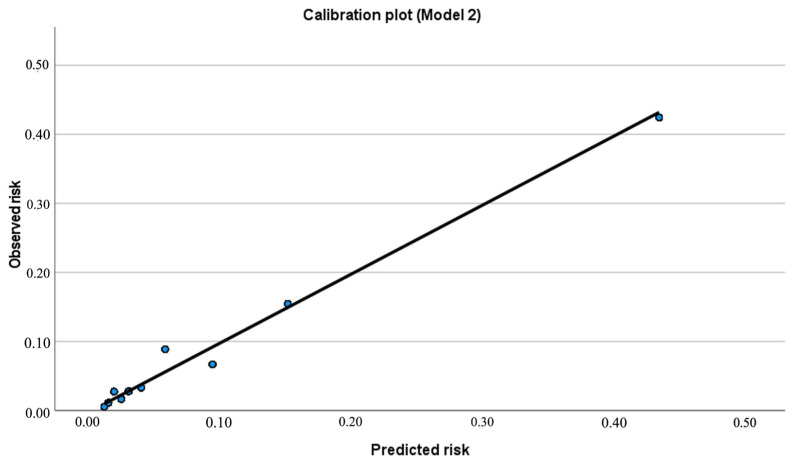
Calibration plot for Model 2.

At the optimal probability threshold (Youden cut-off 0.122), the final model achieved sensitivity 66.2%, specificity 86.2%, PPV 31.1%, NPV 96.5%, and overall accuracy 84.5% (TN = 1417, FP = 226, FN = 52, TP = 102). Calibration was acceptable (Hosmer–Lemeshow χ^2^ = 5.87, df = 8, *p* = 0.662) with an O:E ratio of 1.00, and the calibration plot showed close agreement between predicted and observed risks across deciles (Table 5; Figure 3).

## 4. Discussion

### 4.1. Principal Findings

In this hospital-based cohort of 1797 children aged 2–59 months hospitalized with pneumonia, 8.6% required respiratory support during admission. Children who required support were younger and had substantially higher prevalences of malnutrition, recurrent pneumonia, cyanosis, tachypnea, and chest indrawing. Inflammatory markers derived from routine blood tests differed in biologically plausible directions—higher CRP, higher neutrophil proportion, lower lymphocyte proportion, and higher NLR—while total WBC and MPV were not informative. In multivariable analysis, cyanosis, chest indrawing, malnutrition, recurrent pneumonia, and younger age remained independently associated with respiratory support requirement. Adding NLR improved model discrimination meaningfully (AUC 0.840 vs. 0.754), with acceptable calibration and high NPV, indicating potential utility for clinical triage and early escalation planning.

### 4.2. Comparison with Existing Evidence and Biological Plausibility

Clinical signs and the “hypoxemia problem.” Our strongest bedside predictors—cyanosis and chest indrawing—are consistent with established clinical understanding: they reflect advanced respiratory compromise and increased work of breathing. However, multiple studies emphasize that clinical signs alone are imperfect for detecting hypoxaemia, and that pulse oximetry should be widely implemented for childhood pneumonia care, especially in LMIC settings [3,21]. Global policy documents highlight that hypoxaemia is frequently under-detected among children with pneumonia in low- and middle-income countries, including those without classical danger signs, underscoring the need for routine pulse oximetry and improved access to oxygen therapy [22]. Recent World Health Organization guidelines emphasize that clinical signs and symptoms alone are insufficient for reliable detection of hypoxaemia, and recommend that health facilities caring for children with pneumonia be equipped with pulse oximeters [21]. These data contextualize our results: the very high odds for cyanosis likely reflect “late” clinical hypoxaemia, while the moderate but strong association for chest indrawing aligns with broader evidence that increased work of breathing is closely linked to severe outcomes and escalation needs.

Age and vulnerability in infants. The increased risk observed in younger children is consistent with the known vulnerability of infants due to smaller airway caliber, limited respiratory reserve, and less mature immune responses. Global policy documents emphasize the high public health importance of improving access to oxygen therapy for young children, particularly infants, who represent a priority group for severe pneumonia care in low- and middle-income countries [22].

Malnutrition and recurrent pneumonia. The strong association between malnutrition and respiratory support need is also expected and clinically important. Malnutrition is associated with impaired immune responses, decreased respiratory muscle strength, and worse outcomes in pediatric infections. This is consistent with global policy documents highlighting hypoxaemia and malnutrition as key priority conditions for intervention in childhood pneumonia care in low- and middle-income countries [22]. Recurrent pneumonia likely captures underlying vulnerability—chronic airway disease, immune dysfunction, aspiration risk, or adverse exposures—leading to reduced pulmonary reserve and earlier decompensation during acute infection.

### 4.3. Interpretation of Laboratory Findings and the Role of NLR

Our study found that NLR outperformed WBC and MPV and modestly outperformed CRP and neutrophil% as a single discriminator (AUC 0.705). This is clinically attractive because NLR is cheap, rapidly available, and does not require additional tests beyond a standard CBC. The observed pattern—higher neutrophil% and lower lymphocyte% in those needing support—supports the concept that inflammatory stress and lymphocyte suppression may accompany more severe disease phenotypes. Within the broader pediatric pneumonia literature, recent studies have explored inflammatory markers derived from routine blood tests as potential indicators of disease severity [23]. In parallel, descriptive studies from severe pediatric community-acquired pneumonia cohorts have highlighted a high prevalence of viral–bacterial co-infections, underscoring the biological heterogeneity and inflammatory complexity associated with severe disease phenotypes [24]. While NLR is not disease-specific, its value is that it may add incremental information beyond bedside signs, particularly early in admission when decisions about monitoring intensity and escalation readiness are made.

A key design choice in our multivariable modeling was including NLR rather than neutrophil% and lymphocyte% simultaneously to reduce collinearity; this is methodologically reasonable because NLR summarizes the two components in a single ratio that is less redundant and often more stable.

### 4.4. Prediction Model Performance and Clinical Meaning

Discrimination and calibration. The final model (clinical + NLR) achieved AUC 0.840, which indicates good discrimination for a pragmatic clinical outcome in a heterogeneous real-world cohort. Calibration was acceptable (Hosmer–Lemeshow *p* = 0.662; O:E ≈ 1.00), supporting that predicted probabilities were not systematically over- or under-estimating average risk. Good calibration is essential if the model is to be used for triage thresholds, escalation pathways, or resource allocation.

Why PPV is “low” and NPV is “high.” The model’s PPV (~31%) is limited primarily by outcome prevalence (only 8.6% required support), while NPV (~96.5%) is high. This is a common and expected pattern in prediction problems where the event is relatively infrequent: even strong models will generate many false positives at thresholds chosen to preserve sensitivity. Clinically, this means the model is more suitable for “rule-out/reassurance” (identifying low-risk children who may not need intensive monitoring) or for “risk stratification” (prioritizing closer observation), rather than as a standalone trigger for aggressive interventions.

Threshold selection must match purpose. We reported the Youden threshold for a neutral balance; however, real clinical use often prefers higher sensitivity (lower threshold) if the goal is to minimize missed deterioration, especially where staffing is limited or where delayed escalation leads to harm. Conversely, if oxygen resources are constrained, a higher threshold may be chosen to reduce unnecessary escalation. Previous studies have highlighted substantial heterogeneity in outcomes and predictors of respiratory failure in pediatric pneumonia, underscoring the importance of careful evaluation and reporting of prediction model performance [9,25].

### 4.5. Practical Implications in Vietnam and Similar LMIC Settings

Our predictors are intentionally pragmatic: malnutrition history, recurrent pneumonia history, and bedside respiratory signs require minimal equipment, and NLR is widely available. This makes the approach potentially scalable for triage at admission, especially in settings where pulse oximetry coverage and consistent SpO_2_ documentation remain incomplete. Importantly, contemporary global work argues for expanding pulse oximetry availability and improving oxygen systems as a central child survival strategy [26,27]. In that context, our model is intended as a pragmatic admission-based decision-support tool to assist early triage, prioritization of monitoring intensity, and readiness for escalation. It is not intended to replace clinical judgment or pulse oximetry; rather, it complements existing assessment by integrating routinely available clinical signs with NLR to estimate escalation risk.

Pediatric lower respiratory tract infections are heterogeneous, and escalation needs can differ by etiologic subgroup; for example, clinically relevant differences in disease severity and respiratory support requirements have been reported between RSV and non-RSV bronchiolitis in hospitalized infants, supporting the rationale for multivariable risk stratification approaches [28].

### 4.6. Strengths

This study benefits from a relatively large sample size, clinically meaningful outcome, and reliance on routinely collected predictors. We reported both discrimination and calibration, and we preserved sample size by building the primary model around NLR rather than CRP, which had substantial missingness.

### 4.7. Limitations and Future Directions

Several limitations should be emphasized to avoid over-claiming:

Retrospective single-center design may limit generalizability; practice patterns for oxygen initiation, NCPAP, or ventilation can vary across hospitals and time. Treatment-threshold variability and transportability: Initiation of oxygen therapy may be influenced by institutional protocols and clinician thresholds, which can vary across settings and over time, potentially introducing outcome misclassification. Although we defined the endpoint as the maximum escalation level (oxygen only, CPAP, IMV) to improve clinical specificity and to reflect severity-oriented resource needs, external validation is required to assess transportability across hospitals with different oxygen/CPAP availability, monitoring infrastructure, and escalation pathways.

Outcome definition heterogeneity: “respiratory support” may include oxygen, NCPAP, and invasive ventilation; predictors for each may differ. A composite outcome was intentionally chosen to reflect real-world escalation decisions in resource-limited hospital settings, where oxygen therapy, non-invasive ventilation, and invasive ventilation represent a continuum of respiratory support rather than discrete clinical endpoints. Future analyses separating oxygen-only from non-invasive or invasive respiratory support may further clarify predictor-specific associations. Although respiratory support is a pragmatic escalation endpoint, predictors of oxygen-only support may differ from those for higher-acuity support (CPAP/IMV). In our cohort, we therefore reported outcome granularity (initial modality and maximum level reached, with escalation patterns) to improve interpretability; nonetheless, heterogeneity in escalation pathways across institutions may affect transportability, highlighting the need for external validation and potential recalibration.

Missing data: CRP was available only in a subset of children because testing was not performed systematically in routine care and depended on clinical discretion. The indication pattern is likely mixed—CRP may be preferentially ordered in children perceived as more severe, but also in milder presentations with comorbidities/risk conditions (e.g., malnutrition)—suggesting missingness may not be random. Therefore, CRP-related comparisons should be interpreted cautiously and may not generalize to the full cohort. We did not include CRP in the primary model to avoid reduced sample size and selection bias; instead, the main model focused on routinely available bedside predictors and NLR, while CRP was reported descriptively in the tested subset.

Unmeasured confounding: pathogen data, radiographic severity, prior antibiotics, vaccination status, and SpO_2_ values may improve prediction but were not included (or not consistently recorded). Implementation of pulse oximetry in low-income settings has been associated with reductions in childhood pneumonia mortality, highlighting the importance of early detection of hypoxaemia [29].

Model stability and overfitting: With 154 outcome events and a prespecified primary model including seven predictors, the events-per-variable was approximately 22, which is generally considered acceptable for logistic regression development and reduces concern for severe overfitting; nonetheless, external validation and potential recalibration are required.

In addition, other important clinical management factors that may influence patient outcomes and the level of respiratory support required were not analyzed in this study. These may include the appropriateness and timing of antibiotic therapy, the use of corticosteroids, and other treatment-related interventions during hospitalization. Such management variables could potentially affect disease progression and escalation of respiratory care and should be considered in future studies evaluating predictors of respiratory deterioration.

External validation is required: Before clinical deployment, the model should be validated temporally (later months up to November 2025 as planned) and geographically (another hospital). Previous studies have highlighted substantial heterogeneity and risk of bias in prediction models for respiratory failure in pediatric pneumonia, underscoring the need for rigorous reporting and evaluation [25].

Decision-curve analysis was not performed in the present study. Future work will incorporate decision-curve analysis to quantify net clinical benefit across clinically relevant probability thresholds.

## 5. Conclusions

In this retrospective cohort of 1797 children aged 2–59 months hospitalized with pneumonia, an admission-based model combining bedside clinical features with the neutrophil-to-lymphocyte ratio (NLR) showed good discrimination and acceptable calibration for predicting escalation to in-hospital respiratory support. Key predictors included younger age (<12 months), malnutrition, recurrent pneumonia, cyanosis, chest indrawing, and higher NLR. These findings should be considered exploratory and hypothesis-generating from a single-center retrospective cohort; temporal and geographic external validation and prospective evaluation of clinical impact are required prior to clinical implementation.

## Figures and Tables

**Table 1:** Baseline characteristics of the study population by respiratory support status (*n* = 1797), Outcome: Respiratory support requirement (0 = No, 1 = Yes).

Variable	Overall (n = 1797)	No Support (n = 1643)	Support (n = 154)	*p*-Value
Age (months) median (IQR)	17.0 (9.0–28.0)	17.0 (9.0–28.0)	12.5 (6.0–29.0)	0.012
Male sex, n (%)	1086 (60.4)	993 (60.4)	93 (60.4)	0.991
Malnutrition, n (%)	123 (6.8)	85 (5.2)	38 (24.7)	<0.001
Recurrent pneumonia, n (%)	312 (17.4)	257 (15.6)	55 (35.7)	<0.001
Cyanosis, n (%)	24 (1.3)	6 (0.4)	18 (11.7)	<0.001 *
Tachypnea, n (%)	563 (31.3)	490 (29.8)	73 (47.4)	<0.001
Chest indrawing, n (%)	463 (25.8)	381 (23.2)	82 (53.2)	<0.001
CRP (mg/L), median (IQR) ^†^	5.20 (1.40–18.00)	4.70 (1.26–15.27)	16.98 (4.10–34.00)	<0.001
WBC (×10^9^/L), median (IQR)	11.22 (8.22–14.90)	11.13 (8.39–14.82)	11.61 (7.29–16.55)	0.975
Neutrophils (%), median (IQR)	47.4 (31.5–61.7)	45.7 (30.4–60.2)	60.2 (47.0–72.1)	<0.001
Lymphocytes (%), median (IQR)	38.5 (25.8–52.1)	39.7 (27.1–53.6)	26.2 (16.4–38.6)	<0.001
MPV (fL) median (IQR)	7.60 (7.10–8.20)	7.60 (7.10–8.20)	7.75 (7.30–8.20)	0.088
NLR, median (IQR)	1.21 (0.60–2.35)	1.14 (0.57–2.20)	2.27 (1.21–4.50)	<0.001

Footnote: continuous variables were compared using the Mann–Whitney U test; categorical variables using χ^2^ test or Fisher’s exact test. * Fisher’s exact test was used for cyanosis due to small cell counts. ^†^ CRP was available in 909/1797 children (missing 888): No support n = 812, Support n = 97.

**Table 2:** Univariable association with respiratory support requirement (crude odds ratios), Outcome: Respiratory support requirement (0 = No, 1 = Yes).

Predictor	Crude OR (95% CI)	*p*-Value
Male sex (Male vs. Female)	1.00 (0.72–1.41)	0.991
Age <12 months (vs. ≥12 months)	1.99 (1.43–2.77)	<0.001
Malnutrition (Yes vs. No)	6.00 (3.92–9.20)	<0.001
Recurrent pneumonia (Yes vs. No)	3.00 (2.10–4.28)	<0.001
Cyanosis (Yes vs. No)	36.11 (14.10–92.47)	<0.001 *
Tachypnea (Yes vs. No)	2.12 (1.52–2.96)	<0.001
Chest indrawing (Yes vs. No)	3.77 (2.69–5.28)	<0.001
CRP (per 10 mg/L) ^†^	1.08 (1.02–1.15)	0.013
NLR (per 1 unit)	1.33 (1.25–1.41)	<0.001

*Footnote:* Crude odds ratios (ORs) were estimated using univariable logistic regression. * Fisher’s exact test was used due to small cell counts. Estimates for cyanosis should be interpreted cautiously due to sparse data and wide confidence intervals. ^†^ CRP was available in 909/1797 children (missing 888).

**Table 3:** Multivariable logistic regression models for respiratory support requirement; Model 1 (Clinical-only)–N = 1797; Model 2 (Clinical + NLR)–N = 1797 (primary model).

Predictor	Model 1	*p*	Model 2	*p*
	aOR (95% CI)		aOR (95% CI)	
Age < 12 months (vs. ≥12 months)	1.37 (0.95–1.98)	0.091	2.57 (1.69–3.92)	<0.001
Malnutrition (Yes)	3.78 (2.32–6.14)	<0.001	4.33 (2.56–7.33)	<0.001
Recurrent pneumonia (Yes)	1.74 (1.16–2.60)	0.007	1.82 (1.18–2.81)	0.007
Cyanosis (Yes)	23.11 (8.21–65.07)	<0.001	24.02 (7.41–77.87)	<0.001
Tachypnea (Yes)	1.23 (0.84–1.81)	0.294	1.33 (0.88–2.02)	0.18
Chest indrawing (Yes)	3.31 (2.25–4.87)	<0.001	4.19 (2.73–6.43)	<0.001
NLR (per 1 unit)	-	-	1.49 (1.38–1.60)	<0.001
N used	1797		1797	

Footnote: Adjusted odds ratios (aORs) were estimated using multivariable logistic regression. All binary predictors were coded as 0 = No and 1 = Yes; aORs are presented as “Yes vs. No” (and Age < 12 months vs. ≥12 months). NLR was included to avoid collinearity with neutrophil% and lymphocyte%.

**Table 4:** Discrimination performance: single predictors vs. multivariable models.

Model/Predictor	AUC (95% CI)	*p*-Value
Age	0.352 (0.287–0.417)	<0.001
CRP	0.670 (0.615–0.725)	<0.001
WBC	0.453 (0.385–0.521)	0.13
Neutrophils (%)	0.694 (0.643–0.745)	<0.001
Lymphocytes (%)	0.297 (0.246–0.348)	<0.001
MPV	0.543 (0.481–0.605)	0.168
NLR	0.705 (0.653–0.756)	<0.001
Model 1 (Clinical-only)	0.754 (0.706–0.797)	<0.001
Model 2 (Clinical + NLR)	0.840 (0.805–0.873)	<0.001

Footnote: AUCs were calculated with respiratory support as the positive state (Respiratory support requirement = 1). AUC values < 0.50 indicate an inverse direction of association (i.e., lower predictor values correspond to higher risk). For such predictors, the discrimination in the clinically relevant direction can be expressed as 1 − AUC (e.g., Age: 1 − 0.352 = 0.648; Lymphocytes: 1 − 0.297 = 0.703).

**Table 5:** Classification and calibration of the final model (Model 2: Clinical + NLR; N = 1797); AUC (predicted probability): 0.840 (95% CI 0.806–0.874), *p* < 0.001; Optimal cut-off (Youden): 0.122; Confusion matrix at cut-off 0.122: TN = 1417, FP = 226, FN = 52, TP = 102 (N = 1797).

(A) Discrimination and classification at the optimal cut-off (Youden)	
Metric	Value
Cut-off predicted probability	0.122
Sensitivity	0.662 (66.2%)
Specificity	0.862 (86.2%)
Positive predictive value (PPV)	0.311 (31.1%)
Negative predictive value (NPV)	0.965 (96.5%)
Accuracy	0.845 (84.5%)
(B) Calibration	
Calibration Metric	Value
Hosmer–Lemeshow χ^2^ (df)	5.87 (8)
Hosmer–Lemeshow *p*	0.662
Observed/Expected (O:E) ratio	1.00

Footnote: Classification metrics were calculated at the optimal cut-off determined by the Youden index from the ROC curve of predicted probabilities. Calibration was assessed using the Hosmer–Lemeshow goodness-of-fit test (deciles of predicted risk). The O:E ratio was computed as total observed events divided by the sum of expected events across deciles.

## Data Availability

The data supporting the findings of this study are available from the corresponding author upon reasonable request and with appropriate institutional and ethical approvals.
